# Identification and verification of a prognostic signature based on a miRNA–mRNA interaction pattern in colon adenocarcinoma

**DOI:** 10.3389/fcell.2023.1161667

**Published:** 2023-09-06

**Authors:** Qiwu Zhao, Haosheng Li, Wenchang Li, Zichao Guo, Wenqing Jia, Shuiyu Xu, Sixia Chen, Xiaonan Shen, Changgang Wang

**Affiliations:** ^1^ Department of General Surgery, Ruijin Hospital, Shanghai Jiao Tong University School of Medicine, Shanghai, China; ^2^ Department of Interventional Radiography, Ruijin Hospital, Shanghai Jiao Tong University School of Medicine, Shanghai, China; ^3^ Tongji Hospital, Tongji University School of Medicine, Tongji University, Shanghai, China; ^4^ Department of Gastroenterology, Ruijin Hospital, Shanghai Jiao Tong University School of Medicine, Shanghai, China

**Keywords:** colon adenocarcinoma, miRNAs, prognostic signature, RNA therapy, tissue microarray

## Abstract

The expression characteristics of non-coding RNA (ncRNA) in colon adenocarcinoma (COAD) are involved in regulating various biological processes. To achieve these functions, ncRNA and a member of the Argonaute protein family form an RNA-induced silencing complex (RISC). The RISC is directed by ncRNA, especially microRNA (miRNA), to bind the target complementary mRNAs and regulate their expression by interfering with mRNA cleavage, degradation, or translation. However, how to identify potential miRNA biomarkers and therapeutic targets remains unclear. Here, we performed differential gene screening based on The Cancer Genome Atlas dataset and annotated meaningful differential genes to enrich related biological processes and regulatory cancer pathways. According to the overlap between the screened differential mRNAs and differential miRNAs, a prognosis model based on a least absolute shrinkage and selection operator-based Cox proportional hazards regression analysis can be established to obtain better prognosis characteristics. To further explore the therapeutic potential of miRNA as a target of mRNA intervention, we conducted an immunohistochemical analysis and evaluated the expression level in the tissue microarray of 100 colorectal cancer patients. The results demonstrated that the expression level of POU4F1, DNASE1L2, and WDR72 in the signature was significantly upregulated in COAD and correlated with poor prognosis. Establishing a prognostic signature based on miRNA target genes will help elucidate the molecular pathogenesis of COAD and provide novel potential targets for RNA therapy.

## 1 Introduction

Colon adenocarcinoma (COAD) is the second most common cause of cancer-related deaths worldwide (Sung et al., 2021). According to the latest data from the American Cancer Society in 2022, although COAD mortality has been declining annually for several years, this trend masks an increase in mortality among young adults (Siegel et al., 2022). Meanwhile, the prognosis for advanced COAD cases remains poor, with a 5-year survival rate of only 14%. Approximately 20% of patients with COAD have metastases at early diagnosis, and strategies for identifying early biomarkers are limited (Bidard et al., 2013).

Functional small non-coding RNA molecules (ncRNAs) have been identified in numerous eukaryotes, including the human genome (Anfossi et al., 2018). Current studies show that a large number of ncRNAs play an important role in various biological processes, such as stabilizing RNA molecules, regulating gene expression, and cell proliferation (Czech et al., 2018). The specific mechanism is that each class of small ncRNAs, along with a member of the AGO family of proteins, forms a ribonucleoprotein complex known as an RNA-induced silencing complex (RISC) (Iwakawa and Tomari, 2022). The RISC is directed by the small ncRNAs to bind to the target complementary mRNAs and regulate their expression by interfering with mRNA cleavage, degradation, or translation (Esteller, 2011; Panni et al., 2020). Several different factors can fine-tune the stability of RISC activity from the complementarity of guide–target RNAs to the recruitment of other protein partners to post-translational modification of the RISC itself (Iwakawa and Tomari, 2022). Therefore, microRNAs (miRNAs) and their target genes form a very complex network in the cell (He and Hannon, 2004; Brose et al., 2014). Many studies have shown that miRNAs are abnormally expressed in COAD by targeting cancer-related genes in colorectal cancer cells as oncogenes, tumor suppressors, and cancer markers (Ding et al., 2018).

Over the past several years, bioinformatic approaches and molecular biology methods have identified many conventional and unconventional RISCs binding to target mRNAs not only in the 3'UTR but also in the 5'UTR and ORF (Bartel, 2018). These findings are important for further research in predicting functional target sites of miRNAs and potential misses caused by small ncRNAs (Quevillon Huberdeau and Simard, 2019). To date, the depletion or overexpression of miRNAs is mainly verified to be related to the function of its target, and the dramatic change in miRNAs does not appear to affect the whole process of disease as an intervention target (Iwakawa and Tomari, 2015). Compared to techniques, such as gene editing of target sites, miRNAs use multiple strategies to inhibit protein synthesis through a wide variety of interaction binding sites (Dragomir et al., 2022).

In this study, differential gene screening was performed based on data from patients with COAD, followed by gene annotation and enrichment of related biological processes and regulatory pathways. According to the overlap between the screened differential mRNAs and predicted targets of differential miRNAs, a prognostic model can be established to obtain better prognostic characteristics. Interestingly, the genes regulated by specific miRNAs vary by cancer type. To further explore the therapeutic potential of miRNA as an intervention target for mRNA, we performed immunohistochemical (IHC) analysis and evaluated the expression levels of these genes in the tissue microarray of 100 patients with colorectal cancer. Our study predicted new targets based on miRNA–mRNA interactions that are associated with the development and progression of COAD, providing new insights into the molecular pathogenesis and treatment of colorectal cancer.

## 2 Materials and methods

### 2.1 Data collection

The mRNA and miRNA sequencing data of patients with COAD were downloaded from The Cancer Genome Atlas (TCGA) (https://portal.gdc.cancer.gov/projects/TCGA-COAD). The mRNA expression profile included data on 480 tumor tissues and 41 adjacent non-tumor tissues, while the miRNA expression profile included data on 455 tumor tissues and 10 adjacent non-tumor tissues. Survival information for 440 patients was obtained through the OncoLnc database (http://www.oncolnc.org/). These data were used for the identification of differential expression and the establishment of a prognosis model.

### 2.2 Identification of differentially expressed mRNAs and miRNAs

All mRNAs and miRNAs were annotated in accordance with RefSeq ID, according to the HUGO Gene Nomenclature Committee (HGNC) specifications. Subsequently, mRNA and miRNA expression profiles were normalized using *edgeR* (R package), and false discovery rate (FDR) < 0.05 with |log_2_ fold change (FC)|>1 was set as the criteria for statistically significant differential expression. To predict potential target mRNAs, miRNAs were represented by their mature form (miRs) after the identification of differential expression. Sequences of all mature miRNA in FASTA format were collected from miRBase (https://www.mirbase.org/).

### 2.3 miRNA target prediction

Target gene prediction of differentially expressed mature miRNAs was generated from TargetScan, miRanda, and miRDeep2 databases. The overlapped part of the predicted results of the three databases was considered as possible target genes. The intersection of these genes with differentially expressed mRNAs represented genes in COAD that might be regulated by miRNAs. According to the regulation pattern of the RNA-induced silencing complex, miRNA and mRNA interacted in a negative regulatory manner. Therefore, mRNAs in the opposite direction of the corresponding miRNA expression change were determined as differentially expressed target genes.

### 2.4 Gene Ontology and Kyoto Encyclopedia of Genes and Genomes pathway analyses

Gene Ontology (GO) and Kyoto Encyclopedia of Genes and Genomes (KEGG) pathway analyses were performed to reveal potential functions of differentially expressed target genes using *clusterProfiler* (R package). GO analysis indicated the possible role of differentially expressed target genes in the cellular component (CC), molecular functions (MFs), and biological processes (BPs). KEGG pathway analysis revealed the signaling pathways involved in the regulation of cell function by these genes. The results of the enrichment analysis were visualized in dot plots and bar plots.

### 2.5 Construction of a prognostic predictive model

First, multivariate regression analysis data on all mRNAs were downloaded from the OncoLnc database to obtain prognostic mRNAs independent of age and sex. A prognostic predictive model was then established based on the overlap between these mRNAs and differentially expressed mRNAs. Subsequently, a least absolute shrinkage and selection operator (LASSO)-based Cox proportional hazards regression analysis was conducted to identify a prognostic signature through *penalized* (R package). The optimal lambda was determined according to the result of a maximum cross-validation likelihood calculation for 1,000 cycles. The risk score (RS) of each case was calculated as $\sum \beta_{mRNA}∙{\mathrm{Exp}}_{mRNA}$ , where β indicates the coefficient and Exp indicates the expression level. Cases with a risk score equal to or greater than the median were classified as high risk, otherwise as low risk. The hazards ratio of mRNAs in the model was calculated as $e^{\beta_{mRNA}}$ .

Then, measures of effect and confidence intervals of the aforementioned results were generated using *ggforestplot* (R package). According to the hazard proportion of each gene in the model, the prognostic score of patients in the database was calculated and divided into high- and low-risk groups by the median. The Kaplan–Meier survival curves of the two groups were plotted using *survival* (R package). To estimate the prognosis prediction efficiencies of this signature, the receiver operating characteristic (ROC) curve was plotted, and the area under the curve (AUC) was calculated through *pROC* (P package).

The regulatory interactions between signature mRNAs and their potential regulatory miRNAs were identified via *multiMiR* (R package). The miRNA–mRNA regulatory network was visualized by Cytoscape (https://cytoscape.org/).

### 2.6 Tissue specimens and immunohistochemistry

Tissue samples of 100 pathologically diagnosed primary COAD patients were collected from Ruijin Hospital North Campus, Shanghai Jiao Tong University School of Medicine. Specimens were collected in accordance with institutional protocols. The tumor and adjacent normal tissues were embedded in paraffin and then arranged into tissue microarrays. IHC staining was performed with antibodies against SLC4A4 (Abcam, ab187511), SLCO4C1 (Proteintech, 24584-1-AP), POU4F1 (Abcam, ab81213), DNASE1L2 (Thermo Fisher, PA5-68413), WDR72 (Abcam, ab188518), CELF4 (Abcam, ab171740), LPO (Proteintech, 10376-1-AP), SLC11A1 (Abcam, ab211448), and C7 (Thermo Fisher, PA5-103761) according to a previous description (Zhao et al., 2022a).

## 3 Results

### 3.1 Identification of differentially expressed miRNAs and mRNAs in COAD cells

**FIGURE 1:**
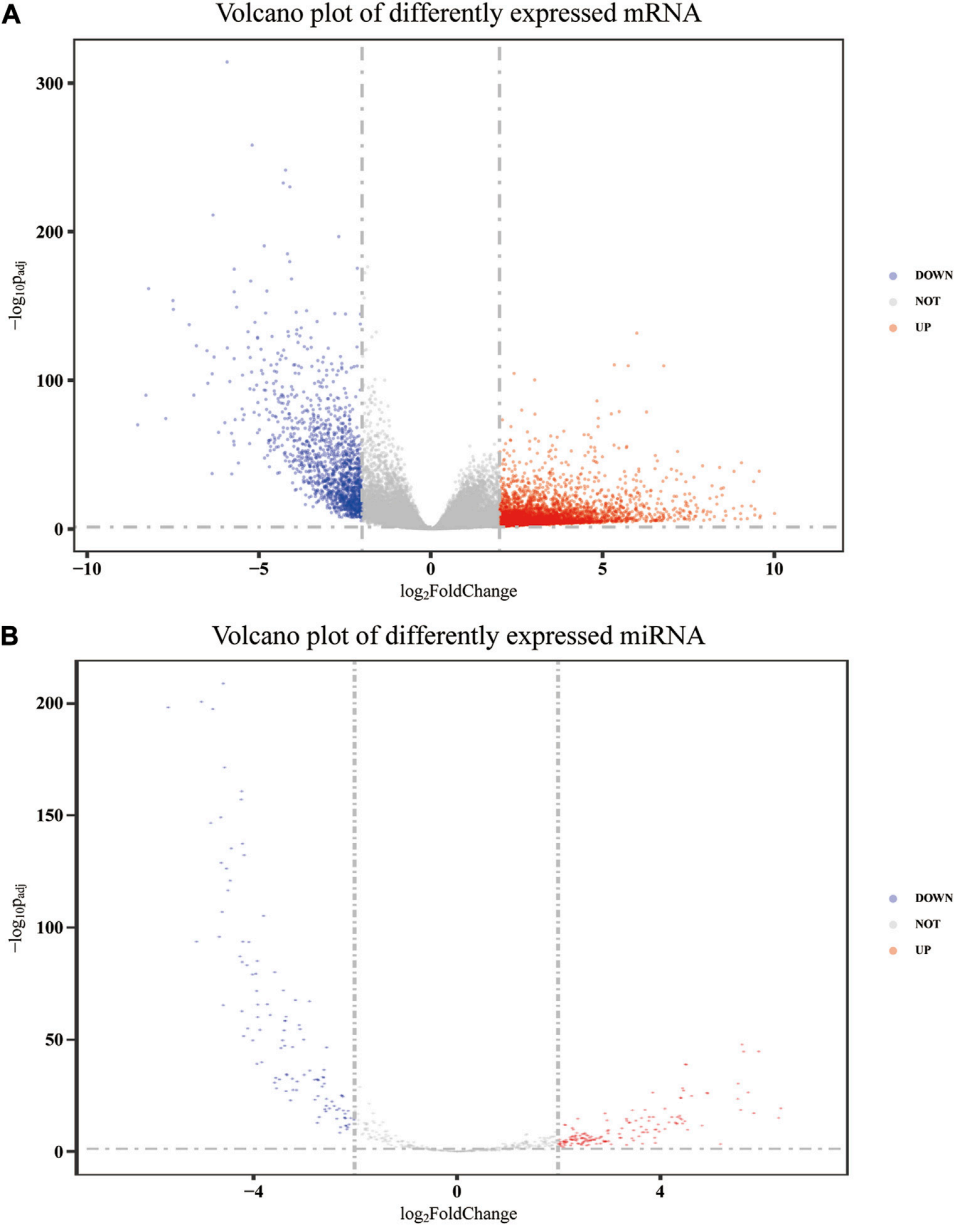
Volcano plot of differently expressed mRNA **(A)** and miRNA precursors **(B)**.

After annotation and removing genes with an average FPKM of less than 1, 32,917, mRNA expression data were obtained from 480 colon adenocarcinoma tumor tissues and 41 adjacent non-tumor tissues, of which 4,447 were upregulated and 1,394 were downregulated. Meanwhile, 581 miRNA precursor expression data were obtained from 455 tumor tissues and 10 adjacent non-tumor tissues. Among these precursors, 270 were differentially expressed, of which 155 were upregulated and 115 were downregulated. After removing those whose mature form was stem-loop and pseudogene, 209 precursors remained. Then, according to the corresponding relationship between the precursor and the mature body, 79 differentially expressed mature miRNAs were determined. Therefore, a total of 5,841 differentially expressed mRNAs and 79 differentially expressed mature miRNAs were identified after data processing. The volcano plot of differently expressed mRNA and miRNA precursors is presented in Figure 1.

### 3.2 Prediction of the target gene of miRNAs and functional enrichment analysis

**FIGURE 2:**
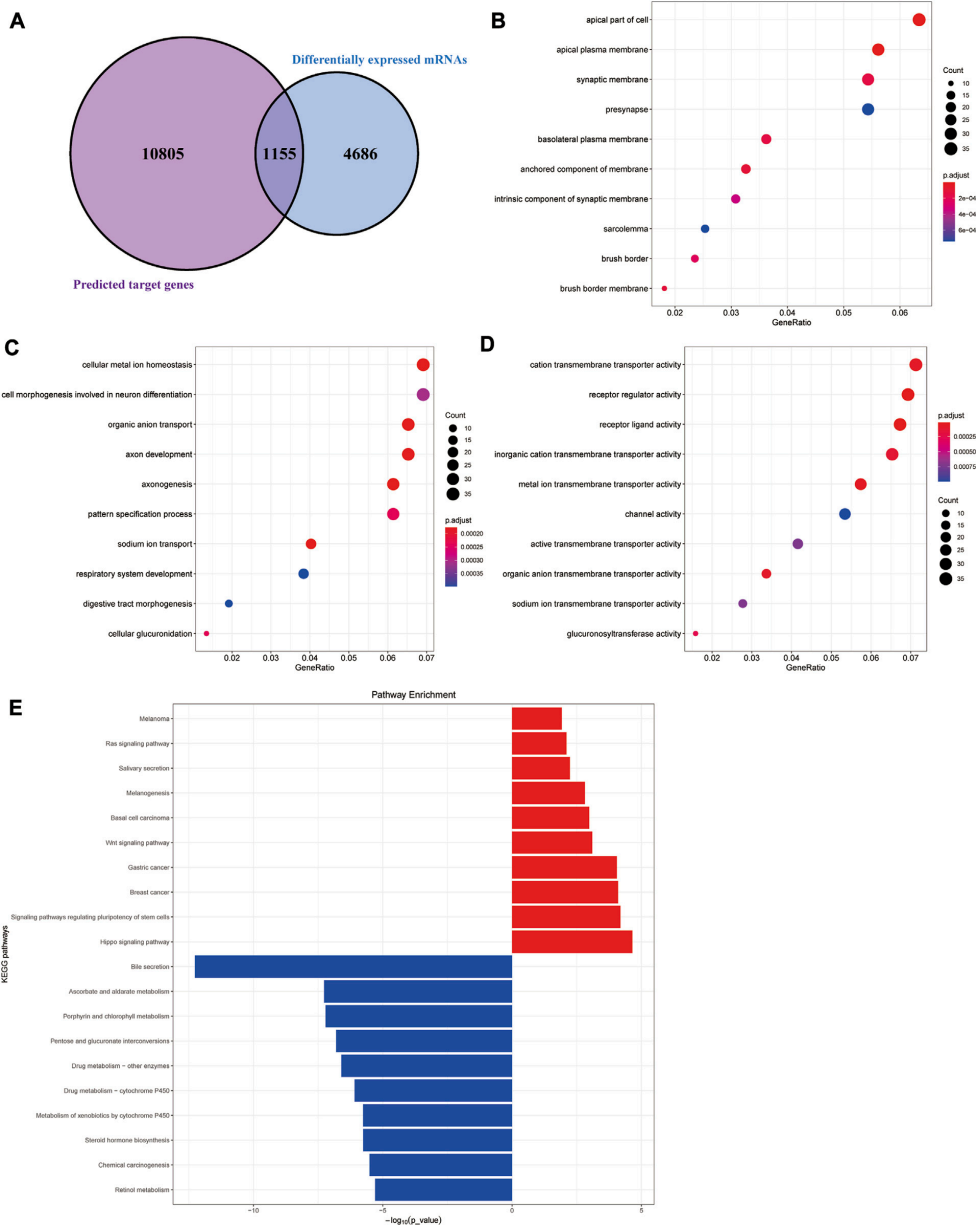
Functional enrichment analyses of differentially expressed target genes. **(A)** Schematic diagram of the process of screening differentially expressed target genes. **(B–D)** Cellular component (CC), biological processes (BP), and molecular functions (MF) results of GO enrichment analysis. **(E)** Results of KEGG pathway enrichment analysis.

The target genes of the aforementioned 79 miRNAs were predicted using TargetScan, miRanda, and miRDeep2 software. After taking the intersection of the aforementioned results, a total of 10,960 predicted target genes were obtained. The intersection analysis of these target genes and differential mRNAs was carried out, and a total of 1,155 potential target genes corresponding to differential miRNAs were identified (Figure 2A). According to the regulation pattern of the RNA-induced silencing complex, 601 mRNAs whose expression changing direction was opposite to that of miRNAs were screened as differentially expressed target genes.

We next performed functional enrichment analysis of differentially expressed target genes. GO enrichment analysis showed that differentially expressed target genes were mainly enriched in the apical part of the cell, apical plasma membrane, and synaptic membrane in the CC; in MFs, they were mainly related to cation transmembrane transporter activity, receptor regular activity, and receptor-ligand activity, and they were mainly involved in several BPs, such as cellular metal ion homeostasis, cell morphogenesis involved in neuron differentiation, and organic anion transport (Figures 2B–D). The results of the KEGG pathway enrichment analysis showed that the upregulated differentially expressed target genes were significantly enriched in classic cancer-related signaling pathways, such as the Hippo signaling pathway, Wnt signaling pathway, and Ras signaling pathway (Figure 2E). In addition, their expression characteristics were highly similar to those of various malignant tumors such as breast cancer, gastric cancer, and basal cell carcinoma. The downregulated differentially expressed target genes were mainly enriched in some metabolism-related pathways, suggesting there were metabolic abnormalities in colon adenocarcinoma.

### 3.3 Establishment and assessment of the target gene-based prognostic signature

To explore the correlation between differentially expressed target genes and patient prognosis, we linked TCGA survival data to mRNA expression data using the OncoLnc database and identified age- and sex-independent risk genes by multivariate Cox regression analysis. Then, we intersected these risk genes with differentially expressed target genes, and a total of 40 prognostic differentially expressed target genes were determined.

**FIGURE 3:**
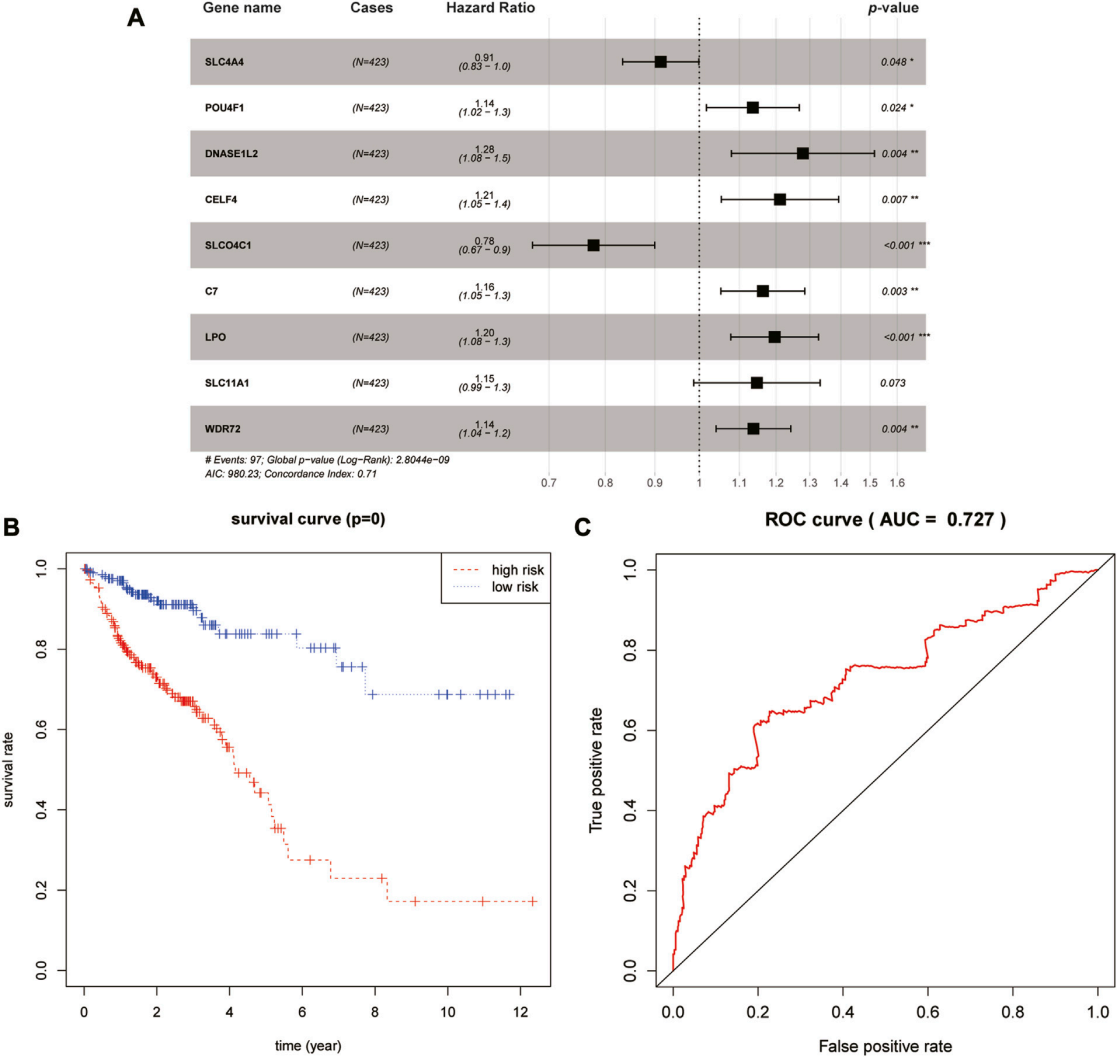
Identification and validation of the prognostic signature. **(A)** Forest plot of the nine-gene signature. **(B)** Kaplan–Meier survival curves of high- and low-risk groups. **(C)** ROC curve of this prognostic signature.

Thereafter, LASSO-based Cox regression was used to establish a prognostic model among the 40 differentially expressed target genes. After LASSO analysis, nine genes were identified as a prognostic signature, namely, SLC4A4, SLCO4C1, POU4F1, DNASE1L2, WDR72, CELF4, LPO, SLC11A1, and C7. A forest plot of the model was generated based on the aforementioned prognostic signatures, and the overall *p*-value of the model was 2.8044e-09 (Figure 3A). After dividing patients into high- and low-risk groups, the Kaplan–Meier survival curves of the two groups demonstrated a significant 5-year survival difference (*p* < 0.05) (Figure 3B). The ROC curve of this signature is shown in Figure 3C. The AUC value of the curve was greater than 0.7, indicating that the model was reliable to some extent.

**FIGURE 4:**
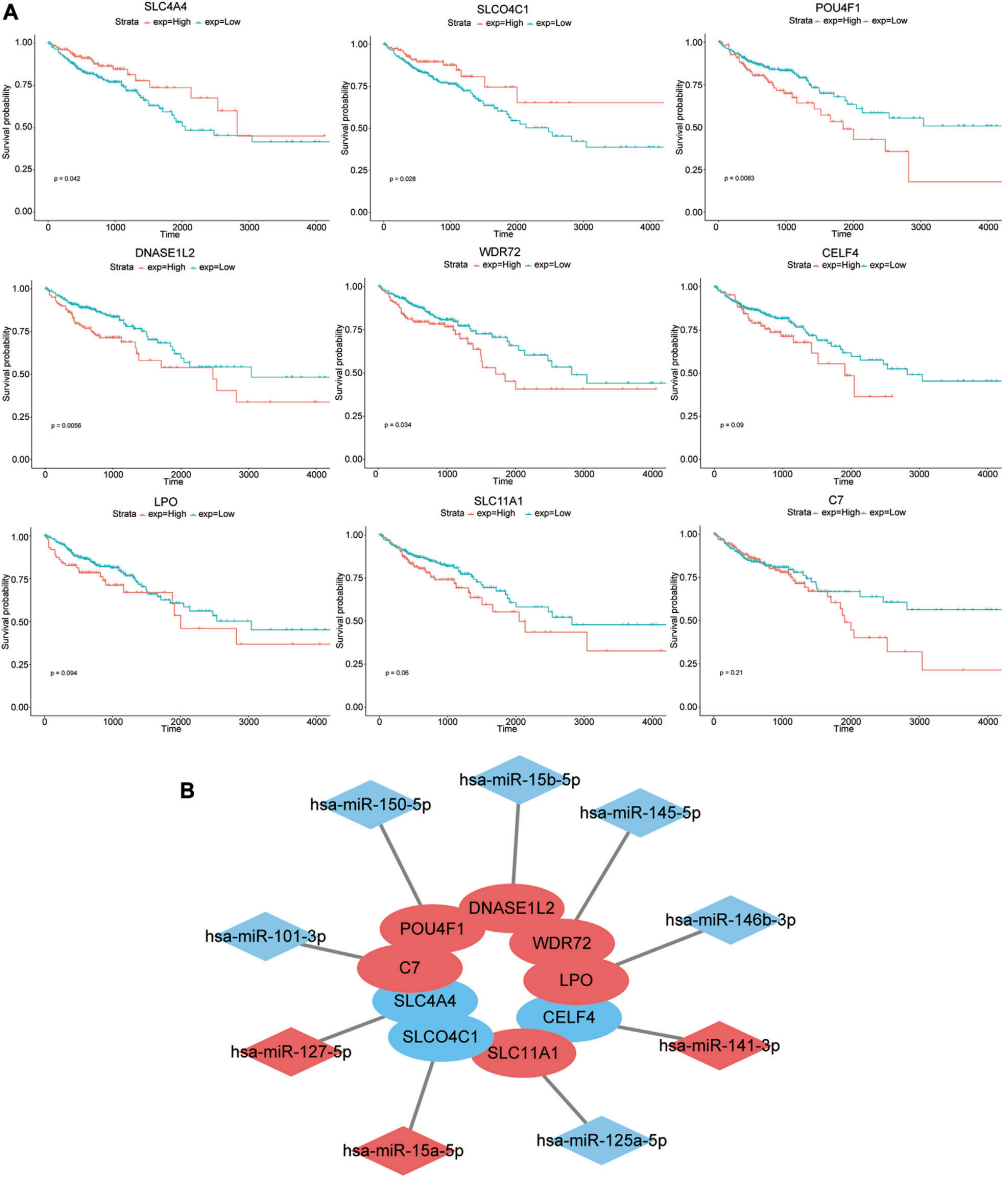
Prognostic effects of nine genes in the model. **(A)** Kaplan–Meier survival curves of high- and low-expression groups of these nine genes. **(B)** Nine pairs of signature genes and potential regulatory miRNAs. Red indicates that the expression level of genes or miRNAs was upregulated in COAD, while blue indicates that the expression level of genes or miRNAs was downregulated.

The prognostic effects of nine genes in the model were further evaluated (Figure 4A). The results suggested that higher expression levels of SLC4A4 and SLCO4C1 in COAD tissues were significantly associated with better prognosis of patients (*p*-values of 0.042 and 0.028, respectively). On the contrary, higher expression levels of POU4F1, DNASE1L2, and WDR72 were significantly correlated with poor prognosis (*p*-values of 0.0083, 0.0056, and 0.034, respectively). The high expression of CELF4, LPO, SLC11A1, and C7 in tumor tissues might be associated with poor prognosis, but they were not statistically significant (*p*-values of 0.09, 0.094, 0.06, and 0.21, respectively).

Furthermore, we evaluated the prognostic effects of specific miRNAs (hsa-miR-127-5p, hsa-miR-15a-5p, hsa-miR-150-5p, hsa-miR-15b-5p, hsa-miR-145-5p, hsa-miR-141-3p, hsa-miR-146b-3p, hsa-miR-125a-5p, and hsa-miR-101-3p) that targeted the aforementioned nine mRNAs. However, there was no significant correlation between their expression level and prognosis (*p* > 0.05) (Supplementary Figure S1A). Nine pairs of signature genes and potential regulatory miRNAs are shown in Figure 4B.

### 3.4 Validation of the prognostic model with clinical specimens

**FIGURE 5:**
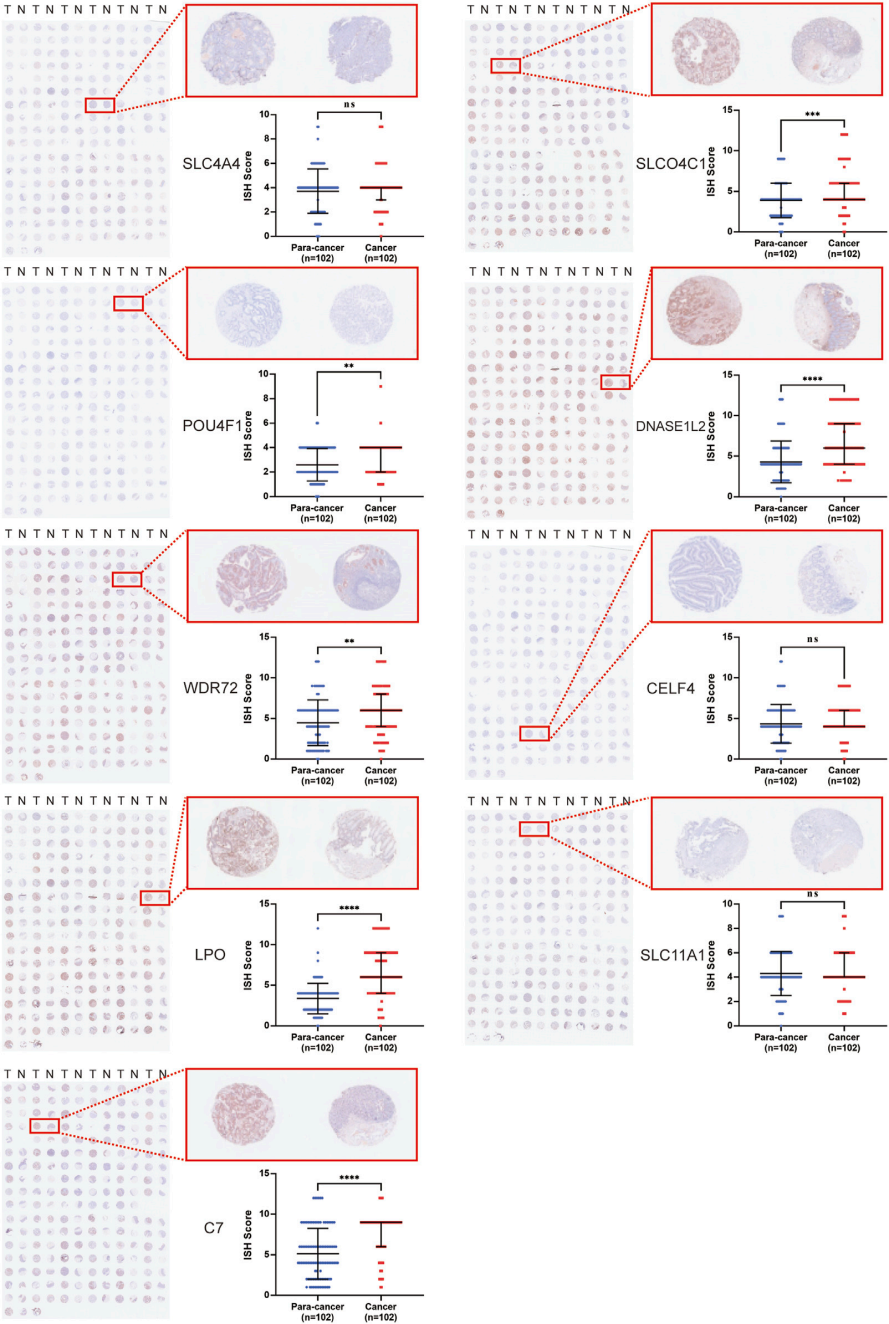
Immunohistochemical staining of tissue microarrays containing cancer and paired para-cancer normal tissues from 100 patients with COAD.

To explore the potential of nine genes in the model as therapeutic targets, immunohistochemical staining of tissue microarrays containing cancer and paired para-cancer normal tissue from 100 patients with COAD was performed, and the expression level of these genes was evaluated. The results revealed that expression levels of SLCO4C1, POU4F1, DNASE1L2, WDR72, LPO, and C7 in tumor tissues were significantly higher than those in normal tissues, while the expression differences of SLC4A4, CELF4, and SLC11A1 were not statistically significant (Figure 5).

In conclusion, the expression level of POU4F1, DNASE1L2, and WDR72 in the signature was significantly upregulated in COAD cells and correlated with poor prognosis. Meanwhile, the expression levels of their corresponding hsa-miR-150-5p, hsa-miR-15b-5p, and hsa-miR-145-5p were higher in tumor tissues than those in adjacent tissues (Supplementary Figure S1B). Thus, these three genes were ideal targets for miRNA therapy.

## 4 Discussion

Due to the heterogeneity among tumors, it is necessary to search in an individualized manner for diagnostic biomarkers and therapeutic target molecules in COAD. In this study, a total of 5,841 differentially expressed mRNAs and 79 differentially expressed mature miRNAs were identified through analysis of the TCGA database. We identified the mRNA and miRNA gene expression characteristics of COAD. Next, screening was conducted according to the pairwise relationship of the reverse regulation of miRNA and mRNA. The results of GO enrichment analysis and KEGG pathway analysis for differential genes were the Hippo signaling pathway, Wnt signaling pathway, and Ras signaling pathway, which is similar to the characteristics of other solid tumors, such as breast cancer and gastric cancer. Abnormal expression of miRNAs is frequently observed in several cancers, and this novel COAD miRNA expression profile will be a useful tool to elucidate the molecular pathogenesis of the disease (Goh et al., 2016; Liu et al., 2021a; Bocchetti et al., 2021).

In order to explore the relationship between differential target gene-based prognosis, a prognostic model with nine characteristics based on a LASSO-based Cox proportional hazards regression analysis was developed, including SLC4A4, SLCO4C1, POU4F1, DNASE1L2, WDR72, CELF4, LPO, SLC11A1, and C7. The AUC value of the ROC curve is also relatively significant. In order to further verify the accuracy and precision of the aforementioned model, we separately analyzed the expression levels of a single mRNA and its paired miRNA in the tumor and paired normal tissue in the database, taking the prognosis into consideration simultaneously. We built immunohistochemical staining of a tissue microarray containing data from 100 patients with colorectal cancer at the protein level and found that POU4F1, DNASE1L2, and WDR72 in the signature were significantly upregulated, and correlated with poor prognosis with a potential therapeutic target strategy. Multiple oncogenic signaling pathways are activated in COAD cells, all of which are regulated by miRNA, and inhibition of signaling pathway activation is an attractive strategy for COAD treatment (Sanchez-Vega et al., 2018; Napolitano et al., 2019; Park et al., 2019; Li et al., 2022).

miRNAs hold promise as disease-related biomarkers in plasma. Some studies seek specific miRNA combinations that can enhance screening sensitivity and specificity, including miRNA biomarkers, at various stages from research to clinical application (Siskova et al., 2020; Ferlizza et al., 2021). For instance, osteomiR and thrombomiR are diagnostic kits developed by TAmiRNA (Austria), based on circulating miRNA profiles (Heilmeier et al., 2016; Chhabra, 2021). The osteomiR kit evaluates fracture risk in patients with osteoporosis by examining the expression profiles of 19 miRNAs. ThrombomiR measures platelet function based on the expression profiles of 11 miRNAs, which is used to evaluate a patient’s susceptibility to developing type 2 diabetes and other cardiovascular diseases. However, these kits are for research purposes and are not clinically approved. The miRview meso and miRview mets assays, proposed by Rosetta Genomics, offer reliable miRNA-based methods for differentiating mesothelioma (cancer caused by asbestos exposure) from other types of lung cancer, using the expression levels of three miRNAs (miR-193-3p, miR-200c, and miR-192) (Benjamin et al., 2010). The miRview mets is a clinical test method based on the expression levels of 64 miRNAs to determine the primary source of the tumor (Meiri et al., 2012).

Current research focuses on discovering specific miRNAs that can enhance screening sensitivity and specificity for colorectal cancer (CRC) biomarkers in plasma. The miR-21 and the miR-29 families (miR-29a, miR-29b, and miR-29c) are overexpressed in CRC and are associated with CRC progression and metastasis (Thomas et al., 2015; Tepus and Yau, 2020). There is evidence suggesting that miR-29a and miR-92a may exhibit a good sensitivity of 69%–89% and a specificity of 70%–89.1% in CRC detection (Marcuello et al., 2019). Moreover, the combined application of miRNAs can improve the detection performance compared to single-miRNA detection. miR-19a and miR-19b are upregulated in the plasma of patients with CRC, and their combination yielded an AUC value of 0.82. The combination of four additional miRNAs, namely, miR-18a, miR-29a, miR-15b, and miR-335, showed promising results in distinguishing between controls and CRC with an AUC value of 0.95 (Herreros-Villanueva et al., 2019). These findings underscore the importance of detection methods involving specific miRNA combinations.

Studies have confirmed the important role of miR-150-5p in colorectal cancer. Xiaoxiang Chen et al. elucidated the mechanism by which lncRNA–ZFAS1 upregulates VEGFA/VEGFR2 and the downstream Akt/mTOR signaling pathway through sponging miR-150-5p, while Xiangfeng Meng et al. revealed the process of the LINC00460/miR-150-5p/mutant p53 feedback loop inducing oxaliplatin resistance (Chen et al., 2018; Meng et al., 2020). In the present study, POU4F1, which paired with miR-150-5p, was a neuro transcription factor that was closely related to AML1/ETO gene fusion in acute myeloid leukemia (Dunne et al., 2010). It might affect the prognosis of patients with COAD through exosomes and the tumor immune microenvironment (Cui et al., 2022).

Chao Liu et al. reported that miR-15b-5p could downregulate PD-L1 at the post-transcriptional level in microsatellite stability (MSS) colorectal cancer, thereby inhibiting tumorigenesis and sensitizing the tumor to anti-PD-1 therapy (Liu et al., 2021b). In addition, miR-15b-5p could also mediate colorectal cancer invasion and migration by targeting ACOX1 (Sun et al., 2017). As the corresponding gene, DNASE1L2 is involved in the breakdown of cell nuclei during keratinocyte formation in epidermal cornification (Fischer et al., 2007). Currently, there is no report on its role in COAD. In breast cancer, DNASE1L2 influenced the growth and metastasis of cancer cells by modulating the epithelial–mesenchymal transformation process (Liu and Meng, 2021).

miR-145-5p was found to act as a tumor suppressor in colon cancer. It inhibited the proliferation and migration of tumor cells by downregulating CXCL1 and ITGA2 (Zhuang et al., 2021). It also inhibited colorectal cancer tumorigenesis by suppressing the EGFR-associated signaling pathway by targeting RHBDD1 (Niu et al., 2019). In the present study, paired gene WDR72 of miR-145-5p was a docking platform protein that possesses no intrinsic enzymatic activity *per se*. However, it was associated with poorer prognosis in patients with COAD, possibly by regulating N6-methyladenosine (m6A) modifications of mRNA (Zhang et al., 2021).

This study aimed to identify potential miRNA biomarkers and therapeutic targets that differ from those found in other COAD studies. Weijie Xue et al. found that based on the characteristics of 18 miRNAs, different tumor mutation load levels of COAD could be effectively predicted and distinguished, suggesting its correlation with immunosuppressive drugs (Xue et al., 2021). Li Peng Jin et al. established a 5-year PS prediction model with AUC = 0.951 and C index = 0.788, which was composed of 17 optimum prognostic signature DERs, showing that the prognosis of the high-risk group was significantly worse (Jin et al., 2020). Yang Cheng et al. revealed a lncRNA-associated ceRNA network dysfunction in COAD (Cheng et al., 2019). Yinling Mao et al. constructed an RS system based on six characteristics (LINC00899, LINC01503, PRKAG2-AS1, RAD21-AS1, SRRM2-AS1, and USP30-AS1) and screened two prognostic clinical characteristics, including pathological staging and RS model status, to establish a nomogram survival model (Mao et al., 2021). However, there is certain heterogeneity among a large number of patients with colorectal cancer. Patients with colorectal cancer can be classified by molecular classification such as CMS in the modeling of prognostic prediction and therapeutic target prediction to increase accuracy (Guinney et al., 2015; Sirinukunwattana et al., 2021). If the verification sample can be expanded and combined with survival analysis to verify the conclusion in the process of modeling, the accuracy will also be increased.

The advantage of RNA-based gene therapy is that the therapeutic RNA works within the target cell without eliciting an unwanted immune response (Wang et al., 2021). It is worth mentioning that the US Food and Drug Administration (FDA) approved the first siRNA drug in 2018, and small ncRNA drugs targeting various diseases will continue to appear on the market. miRNA mimics and siRNA are highly similar synthetic dsRNAs through the same cellular RNAi pathway. Both are about 22 nt in length as RISC components, which rely on Dicer enzyme processing and AGO family proteins (Rupaimoole and Slack, 2017). Compared to siRNA, reports on miRNA mimic design and chemical modification are few. miRNAs are endogenous post-transcriptional gene regulators that are conserved across species (Setten et al., 2019). miRNA-induced inhibition of the target gene is relatively modest, but the functional effect is powerful due to the synergistic inhibition of multiple targets in integrated cell signaling pathways (Paunovska et al., 2022). RISCs recruit miRNAs into complementary mRNA sequences for bidirectional regulation by miRNA mimics (designed to increase native miRNA activity) and anti-miRNAs (designed to decrease native miRNA activity), which have been studied in animal models and used in clinical trials (Hanna et al., 2019). A phase I clinical trial investigated the use of MRX34, which uses liposomes to deliver a double-stranded miRNA-34a mimic, for the treatment of advanced solid tumors (Hong et al., 2020). In addition, endogenous small ncRNAs, especially miRNAs, can affect the efficiency and stability of mRNA-based translation, bringing more hope and the possibility to realize the full potential of RNA-based therapeutics.

The large-scale regulatory network composed of competitive endogenous RNAs (ceRNA) forms a gene regulatory pattern. The lncRNA–miRNA–mRNA axis plays a crucial role in patients with colorectal cancer, revealing new molecular mechanisms for the development of the disease (Salmena et al., 2011). Wang et al. found that miR-17-5p, miR-125b-5p, miR-129-5p, miR-193a-3p, miR-206, miR-212-3p, miR-363-3p, miR-425-5p, and miR-455-5p are downregulated in patients and associated with longer survival time, potentially indicating a positive prognosis (Xiang et al., 2022). Xie et al. identified two potential exosome regulation axes through differentially expressed exosome gene clusters in patient plasma, namely, lncRNA G016261/miR-150-5p/RBM48 and lncRNA XLOC011677/miR-10b-5p/BEND3 (Zhao et al., 2022b). Zhang et al. used systems analysis and found that the expression of miR-193a-5p, miR-485-5p, miR-17-5p, and miR-92a-3p affects the occurrence and development of CRC. The diagnostic model built on this basis may help improve the diagnostic efficiency for patients with CRC (Meng et al., 2022). In addition, Wang et al. built a prognostic model of the ceRNA network and infiltrating immune cells, which includes genes, such as lncRNA H19/hsa-miR-29b-3p, that have potential prognostic value and identifies the relationship between the infiltrating immune cell prognostic models (Guo et al., 2021). Although numerous studies have emerged on the ceRNA mechanism, revealing that lncRNA could be an effective prognostic biomarker, the complex networks formed by lncRNAs offer additional complexity for the treatment of CRC. However, much of our understanding of the ceRNA network is still in the early stages, and the precise mechanisms and factors related to cancer progression are largely unknown. More advanced whole-genome algorithms for ceRNA prediction are required to identify new prognostic biomarkers. Concurrently, new therapeutic strategies need to be developed, such as new delivery methods for siRNA or CRISPR, to target these ceRNAs (Wang et al., 2019).

Currently, the therapeutic approach for mRNA–miRNA interactions has progressed from research to clinical application, and the stability of miRNA has also been recognized. It is found that over 60% of human protein-coding genes are potential targets of miRNA, highlighting the importance of miRNA–mRNA interactions in diseases (Bartel, 2009; Friedman et al., 2009; Salmena et al., 2011). Interestingly, many miRNA-mediated regulations are moderate, inhibiting target protein expression by less than two-fold and usually without any consequences (Selbach et al., 2008; Seitz, 2009). Extensive research on the miRNA–mRNA regulatory axis will provide an important blueprint for the treatment and prognosis assessment of patients with CRC.

## Data Availability

The original contributions presented in the study are included in the article/Supplementary Material; further inquiries can be directed to the corresponding authors.

## Supplementary material

SUPPLEMENTARY IMAGE S1 Supplementary Figure S1.

SUPPLEMENTARY FIGURE S1 Kaplan–Meier survival curves of high- and low-expression groups of nine miRNAs **(A)**. The expression level of significant miRNAs in COAD **(B)**.
