# Supplementary figures and images for: Identification and verification of a prognostic signature based on a miRNA–mRNA interaction pattern in colon adenocarcinoma
Source: Front Cell Dev Biol. 2023 Sep 6;11:1161667. doi: 10.3389/fcell.2023.1161667 (PMC10511881; doi:10.3389/fcell.2023.1161667)

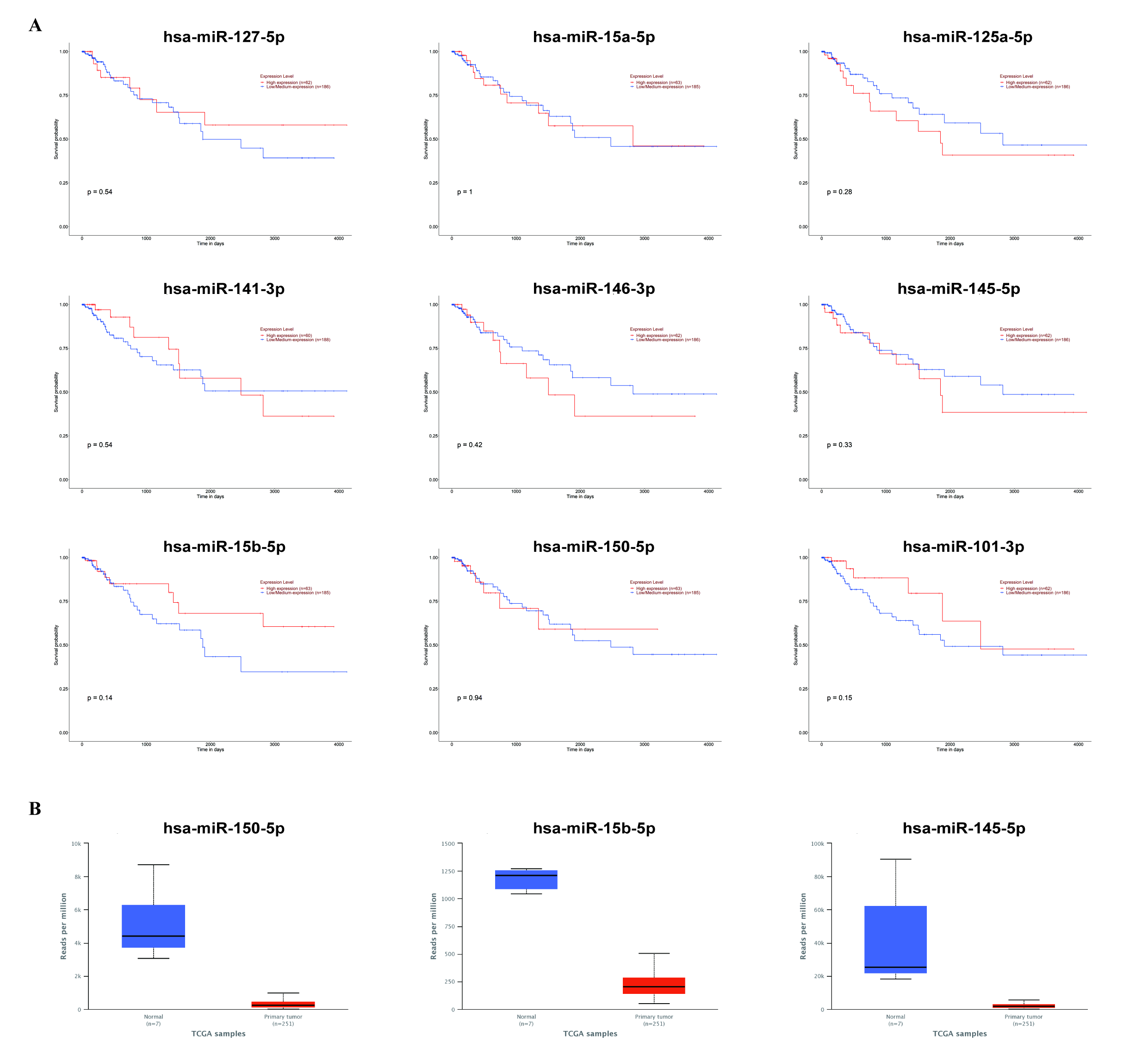

Supplement: Supplementary file 1 [file Image1.tif]
